# Immunology and Cell Biology of Parasitic Diseases 2013

**DOI:** 10.1155/2013/101268

**Published:** 2013-06-17

**Authors:** Luis I. Terrazas, Abhay R. Satoskar, Miriam Rodriguez-Sosa, Jorge Morales-Montor

**Affiliations:** ^1^Unidad de Biomedicina, Facultad de Estudios Superiores-Iztacala, Universidad Nacional Autónoma de México, Avenida de Los Barrios No. 1 Los Reyes Iztacala, 54090 Tlalnepantla, Edo. De MEX, Mexico; ^2^Departments of Pathology and Microbiology, Starling Loving Hall M418, The Ohio State University, Columbus, OH 43210, USA; ^3^Departamento de Inmunología, Instituto de Investigaciones Biomédicas, Universidad Nacional Autónoma de México, Circuito Escolar S/N Ciudad Universitaria, 04510 México, DF, Mexico

In this, our third, special issue we have chosen 19 reports, including both original and review papers, which represent 60% of the papers submitted to this new special issue. This is a sample that we have maintained the quality of the submissions as an essential requirement to get published in this new era of BioMed Research International (formerly Journal of Biomedicine and Biotechnology).

This special issue was supported mainly by some of the attendants to our V Mexican Immunoparasitology meeting, which has been continuously holding every two years since starting in 2004. This event was created with the expectation to meet Mexican scientist and overseas guest scientists interested in the field of immunoparasitology, given that every time is more difficult to find right places on parasitological congresses as well as in immunological congresses to discuss this interface of knowledge, which has great importance.

Infections by parasites, starvation, insufficient shelter, and lack of clean water sources are the greatest barriers to health in our world's growing population. Several of the parasitic infections that are very common throughout the world frequently occur with mild, obscure symptoms or none at all. It is common for a host to be asymptomatic, which makes more difficult both the diagnostic and the on time treatment. Parasitic infections that are thought to be the most prevalent worldwide include toxoplasmosis, ascariasis, toxocariasis, hookworm disease, amoebiasis, and trichomoniasis; many of them are discussed in this special issue. The scope of this topic could be immense, as there are hundreds of parasitic species that infect humans, and also because parasitic infections are considered as one of the leading causes of high morbidity and mortality in underdeveloped countries, mainly in children and the elderly, affecting the physical and intellectual capabilities of such population and resulting in high expenses in treatment and rehabilitation. Moreover, in recent years the massive migration of people from the south countries, where parasitic diseases are common, towards developed countries fueled that many parasitic infections are now reemerging and generating new health problems at a global level.

The knowledge about parasitic diseases at different levels, such as life cycles, structures, molecular biology, development of new drugs and vaccines, and its ability to escape or modulate different mechanisms of defense of its hosts, is essential to better development of therapies and diagnostics. This special issue addresses many of such issues. We have divided the special issue in two blocks, the first dealing with protozoan parasites and the second one dealing with different helminth infections. In both you will find new and important information related to parasitic diseases.

In our first block (protozoan infections) we started with a very opportune review by A. Vazquez-Mendoza et al. “*Parasitic infections: a role for C-type lectins receptors*” where they describe how important, as well as complicated, is the early recognition of parasites by C-type lectin receptors (CLRs), which are expressed by distinct subsets of dendritic cells and macrophages. They address the question: how are CLRs involved in both pathogen recognition and the internalization of parasites? They conclude that parasite recognition through different carbohydrates localized on its surface or in the excretory/secretory products are essential to the outcome of the infection, and this seems as a new opportunity area to block the entrance or increase the immune response against them. Next, we have a couple of papers related to amoebiaisis studies: first M. Omaña-Molina et al., propose a new role for the proteases from *Acanthamoeba* in the tissue invasion in “*Reevaluating the role of acanthamoeba proteases in tissue invasion: observation of cytopathogenic mechanisms on MDCK cell monolayers and hamster corneal cells*”. As they remark, the most important observation in this study was the fact that proteases apparently do not participate in tissue destruction. By challenging the paradigm, they found that no lysis of corneal tissue was observed as it was previously suggested. Thus, their results support the notion that the invasion and disruption of corneal tissue during acantamoebiasis is performed by the penetration of the amoebae through cell junctions, either by the action of proteases promoting cellular separation but not by their destruction and/or a mechanical effect exerted by amoebae. In the second one H. Aguilar-Diaz et al. used new technology tools to demonstrate that silencing an enzyme in *Entamoeba histolytica* avoids the formation of cysts. This is very important finding since encystment is an essential process in the biological cycle of the human parasite *E. histolytica*; these findings may allow to develop new control strategies against this parasite as stated in “*Silencing of Entamoeba histolytica glucosamine 6-phosphate isomerase by RNA interference inhibits the formation of cyst-like structures*”.

Trypanosomatids parasites are a group of protozoan that importantly affects humans. In this area C. Lugo-Caballero et al. in “*Identification of protein complex associated with LYT1 of Trypanosoma cruzi*” identified a protein complex associated with LYT1 in *Trypanosoma cruzi*. This is important because few molecules have been reported participating in the intracellular phase of *T. cruzi* life cycle. Their approach led them to identify the LYT1 interaction profile, thereby providing insights into the molecular mechanisms that contribute to parasite stage development and pathogenesis. In another study, J. A. Díaz-Gandarilla et al. “*PPAR activation induces M1 macrophage polarization via cPLA2-COX-2 inhibition, activating ROS production against Leishmania Mexicana*” showed that PPAR activation induces M1 macrophage polarization via cPLA2-COX-2 inhibition, thus, activating ROS production against *Leishmania mexicana*. By doing a very thorough analysis, they conclude that PPAR agonists used in their work induce M1 macrophages polarization via inhibition of cPLA2 and the increase of aggressive microbicidal activity via reactive oxygen species production. To finalize with the collaborations in this topic, Y. Flores-García et al. “*CD4* ^+^ *CD25* ^+^ *FOXP3* ^+^ *Treg cells induced by rSSP4 derived from T. cruzi amastigotes increase parasitemia in an experimental chagas disease model*” discuss the role in Treg cells induced by rSSP4 derived from *T. cruzi* amastigotes have in increasing parasitemia in an experimental Chagas' disease model. As authors state, currently, there is a considerable controversy on the role of Treg cells during *T. cruzi* infection, the main point being whether these cells play a negative or a positive role. They found that the adoptive transfer of CD4^+^ CD25^+^ FOXP3^+^ T cells from rSSP4- (a recombinant *T. cruzi* amastigote derived protein) reduces cardiac inflammation and prolongs hosts' survival but at the same time, increases blood parasitemia and parasite loads in the heart.

Trichomoniasis is one of the most common sexually transmitted infections in the world; in this issue F. J. Rendón-Gandarilla et al., by studying *Trichomonas vaginalis* cytoadherence, found that the TvLEGU-1, a legumain-like cysteine proteinase, plays a key role in pathogenesis of this parasite.

In our second section, related with helminth infections, several authors discuss relevant issues associated with these widely distributed infections.

B. Faz-López et al. in “*Signal transducer and activator of transcription factor 6 signaling contributes to Control host lung pathology but favors susceptibility against Toxocara canis infection*” found that the signal transducer and activator of transcription factor 6 signaling contributes to control host lung pathology during *T. canis *infection. However, though it helps in the lung, it does favor to increase the susceptibility against this widely distributed parasite. Their findings demonstrate that a Th2-like response induced via STAT6-mediated signaling pathway mediates susceptibility to larval stage of *T. canis*. Furthermore, they also indicate that unlike most gastrointestinal helminths, immunity against larvae of *T. canis* is not mediated by a Th2-dominant response.

*Taenia crassiceps* is a cestode parasite of rodents (in its larval stage) and canids (in its adult stage) that can also parasitize immunocompromised humans. In the case of the hygiene hypothesis, A. M. Ortiz-Flores et al. in “*Taenia crassiceps infection does not influence the development of experimental rheumatoid arthritis*” found that *T. crassiceps* infection does not influence the development of experimental rheumatoid arthritis. On the same line, A. N. Peón et al. in “*Immunoregulation by Taenia crassiceps and its antigens*” discuss the potential role that antigens from *T. crassiceps* have as immunoregulators. The authors have discovered that in mice and human cells, the whole parasite or its antigens have a strong capacity to induce chronic Th2-type responses that are primarily characterized by high levels of Th2 cytokines, low proliferative responses in lymphocytes, an immature and LPS-tolerogenic profile in dendritic cells, the recruitment of myeloid-derived suppressor cells, and, specially, alternatively activated macrophages. Thus, they thoroughly review the work of others and themselves in regard to the immune-modulation induced by *T. crassiceps* and its antigens and compare their advances in the understanding of this parasitic infection model with the knowledge that has been obtained from other selected models. Moreover, L. I. Terrazas et al. in the paper “*Helminth excreted/secreted antigens repress expression of LPS-induced let-7i but not miR-146a and miR-155 in human dendritic cells*” have found that helminth excreted/secreted antigens of *T. crassiceps* repress the expression of LPS-Induced Let-7i but not miR-146a and miR-155 in human dendritic cells. Since microRNAs have emerged as early key regulators of immune responses due to their influence on immune cells' function and probably the outcome of several infections. Currently, it is largely unknown if helminth parasites and their antigens modify host microRNAs expression. This let-7i downregulation in dendritic cells constitutes a novel feature of the modulatory activity that helminth-derived antigens exert on their host.

Macrophages are also critically involved in the interaction between *T. crassiceps* and the murine host immune system. Also, a strong gender-associated susceptibility to murine cysticercosis has been reported. Thus, C. Togno-Pierce et al. in “*Sex-associated expression of Co-stimulatory molecules CD80, CD86, and accessory molecules, PDL-1, PDL-2 and MHC-II, in F480+ macrophages during murine cysticercosis*” examined the sex-associated expression of MHC-II, CD80, CD86, PD-L1, and PD-L2 expressed on peritoneal F4/80+ macrophages of BALB/c mice exposed to *T. crassiceps* total extract. They found that female mice recruited higher number of macrophages to the peritoneal cavity than males. Furthermore, macrophages from infected animals showed increased expression of PDL2 and CD80 that was dependent from the host gender. Their findings suggest that macrophage recruitment at early time points during *T. crassiceps* infection is a possible mechanism that underlies the differential sex-associated susceptibility displayed by the mouse gender.

Schistosomiasis, caused by infection with *Schistosoma* species, remains an important parasitic zoonosis. In the design of a better DNA vaccine against the human trematodes species of *Schistosoma*, Y. Cao et al. in “*Gene gun bombardment with DNA-coated golden particles enhanced the protective effect of a DNA vaccine based on thioredoxin glutathione reductase of Schistosoma japonicum*” developed a gene gun bombardment with DNA-coated golden particles that enhanced the protective effect of a DNA vaccine based on a thioredoxin glutathione reductase of *Schistosoma japonicum*, which was very effective when tested in vivo.

In the field of biotechnology, G. Peña et al. in “*In vitro ovicidal and cestocidal effects of toxins from Bacillus thuringiensis on the canine and human parasite Dipylidium caninum*” showed that toxins from *B. thuringiensis* have a strong in vitro ovicidal and cestocidal effects on the parasite *Dipylidium caninum* which causes an important zoonosis in dogs and humans. *B. thuringiensis* is a gram-positive soil-dwelling bacterium that is commonly used as a biological pesticide. Authors remark that this bacterium may also be used for biological control of helminth parasites in domestic animals. They found that proteins of the strain GP526 of *B. thuringiensis* directly act upon *D. caninum* showing ovicidal and cestocidal effects. Thus, *B. thuringiensis* is proposed as a potential biological control agent against this zoonosis.

T. Çelik et al. in the paper “*Toxocara seroprevalence in patients with idiopathic Parkinson's Disease: chance association or coincidence?*” studied specific IgG antibodies against *T. canis* in 50 patients with idiopathic Parkinson and 50 healthy volunteers. They investigated the clinical history of three patients infected with *T. canis*. They also studied specific IgG antibodies against *Toxoplasma gondii* in these groups. Antibodies anti-*Toxocara* were found in 3 idiopathic PD (6%) and antibody titer was not found in control. A patient had history of the presence of dog and current dog ownership. They did not detect any statistically significant association between *T. canis* and IPD. But, we believe that further comprehensive studies are required for understanding whether there is a causal relation between toxocariasis and IPD. In another nonrelated topic to parasitology, but very important to immunobiology, M. G. R. García and F. G. Tamayo in “*The importance of the nurse cells and regulatory cells in the control of T lymphocyte responses*” discuss the Importance of the nurse cells in the control of T lymphocyte responses. The modulatory role that neurotransmitters and hormones play in these interactions is also revised in their contribution. F. Alba-Hurtado and M. A. Muñoz-Guzmán in “*Immune responses associated with resistance to haemonchosis in sheep*” review the immune responses associated with resistance to haemonchosis in sheep. Their contribution examines the actual known on immunological and genetic factors associated with sheep resistance to infection by *Haemonchus contortus*. Interestingly, such resistance appears to be an inheritable genetic trait.

Human neurocysticercosis by *Taenia solium* is considered an emergent severe brain disorder in developing and developed countries. Discovery of new antiparasitic drugs has been recently aimed to restrain differentiation and establishment of the *T. solium* adult tapeworm, for being considered a central node in the disease propagation to both pigs and humans. On regard to new parasiticidal drugs against *T. solium*, R. Hernández-Bello et al. in “*A new parasiticidal compound in T. solium cysticercosis*” reported a new parasiticidal compound. They tested the effect that 16*α*-bromoepiandrosterone (EpiBr), a dehydroepiandrosterone (DHEA) analogue, had on the cysticerci of *T. solium* in both in vitro and in vivo studies. Authors showed that in vitro treatment of *T. solium* cultures with EpiBr reduced scolex evagination, growth, motility, and viability in a dose- and time-dependent fashion. Administration of EpiBr prior to infection with *T. solium* cysticerci in hamsters reduced the number and size of developed tapeworms in the intestine, compared with controls. These effects were associated with an increase in splenocyte proliferation in infected hamsters. These results leave open the possibility of assessing the potential of this hormonal analogue as a possible antiparasite drug, particularly in cysticercosis and taeniosis. Finally, G. Escobedo et al. in “*Tamoxifen treatment in hamsters induces protection during taeniosis by Taenia solium*” demonstrated that tamoxifen (an anti-oestrogen) treatment in hamsters induces protection during taeniosis by *Taenia solium*. Their results showed that tamoxifen inhibited evagination of *T. solium* cysticerci in a dose-time-dependent manner. In vivo, administration of tamoxifen to hamsters decreased the intestinal establishment of the parasite by 70%, while recovered tapeworms showed an 80% reduction in length, appearing as scolices without strobilar development. Since tamoxifen did not show any significant effect on the proliferation of antigen-specific immune cells, intestinal inflammation, and expression of Th1/Th2 cytokines, in spleen and duodenum, this drug could exert its antiparasite actions by having direct detrimental effects upon the adult tapeworm.

We hope our readers find this third special issue of enticing and enjoy reading contributions by all authors.

